# Burden and Needs of Patients with Severe GvHD from the Supportive and Palliative Care Perspective—A Literature Review

**DOI:** 10.3390/cancers13112697

**Published:** 2021-05-30

**Authors:** Freya Wenzel, Anne Pralong, Udo Holtick, Christoph Scheid, Marco Herling, Steffen T Simon

**Affiliations:** 1 Department of Palliative Medicine, University Hospital and Faculty of Medicine, University of Cologne, 50937 Cologne, Germany; freya.wenzel@uk-koeln.de (F.W.); anne.pralong@uk-koeln.de (A.P.); 2 Center for Integrated Oncology Aachen-Bonn-Cologne-Duesseldorf (CIO ABCD), University Hospital and Faculty of Medicine, University of Cologne, 50937 Cologne, Germany; udo.holtick@uk-koeln.de (U.H.); c.scheid@uni-koeln.de (C.S.); marco.herling@uk-koeln.de (M.H.); 3 Department of Internal Medicine I, University Hospital and Faculty of Medicine, University of Cologne, 50937 Cologne, Germany; 4 Clinic of Hematology, Cellular Therapy, Hemostaseology, University of Leipzig, 04107 Leipzig, Germany; 5 Center for Health Services Research (ZVFK), University Hospital and Faculty of Medicine, University of Cologne, 50933 Cologne, Germany

**Keywords:** graft-versus-host disease, palliative care, supportive care, hematopoietic stem cell transplantation, quality of life, physical function

## Abstract

**Simple Summary:**

Patients who have been treated with an allogeneic, hematopoietic stem cell transplantation can develop severe graft-versus-host disease. This complication may place patients in a life-threatening situation, in which a curative goal of care can no longer be achieved and needs to be changed into a palliative one. In our clinical experience, this patient group is very heterogenous, with a high disease burden and special needs that are often overlooked. In this review, we summarize the current literature on the needs and burdens of patients with severe forms of graft-versus-host disease from a supportive and palliative care perspective to draw a comprehensive picture of this patient group. Despite a fundamental lack of studies, the findings suggest that the more severe the GvHD, the worse the quality of life and physical functioning. The relative void of data highlights the need for research on this special issue in order to optimize the treatment and care of patients with severe graft-versus-host disease.

**Abstract:**

Graft-versus-host disease (GvHD) is a frequent, and often life-threatening, complication after an allogeneic, hematopoietic stem cell transplantation (allo-SCT). It can appear in an acute or a chronic form and presents different grades of severity. Particularly, the severe forms of GvHD are often responsible for a change of the curative intent for allo-SCT into a palliative goal of care. For this non-systematic review, we conducted a focused literature search in the MEDLINE database via PubMed to examine whether patients with severe forms of GvHD might have special needs and burdens from a supportive and palliative care perspective. To draw a comprehensive picture of this patient group, we included findings on quality of life (QoL) and physical symptoms and function as well as psychological and spiritual well-being. In most domains, patients with severe forms of GvHD showed greater impairment and a higher symptom burden compared to patients with milder forms of GvHD. However, we could not identify any studies that specifically investigated patients with severe forms of GvHD. Further research in this field is necessary to guarantee the highest standard of care for this very special patient group.

## 1. Introduction

Graft-versus-host disease (GvHD; transplant-versus-recipient reaction) is an immunological reaction that can occur as a result of an allogeneic bone marrow or blood stem cell transplantation (allo-SCT). Acute (a) GvHD is characterized by a rapid onset of clinical manifestations, usually within the first 2 months after allo-SCT, and mainly affects the skin, gastrointestinal tract (GI) and liver [1]. In addition, a distinction is made between late onset aGvHD, occurring after day +100 post-allo-SCT, and recurrent, or persistent, aGvHD [2]. In aGvHD, cells of the innate immunity and donor-specific lymphocyte subpopulations react against tissue-specific structures of the recipient [3].

In chronic (c) GvHD, persistent alloimmune processes lead to tissue and organ inflammation and as a result, to varying degrees of fibrosis and sclerosing processes in various organ systems [4]. Clinically, skin changes as well as mouth and liver affection dominate [2]; however, nearly every organ including the upper and lower gastrointestinal tracts, eyes, lungs, joints or fascia and the genital tract can be affected [5].

GvHD is the leading cause of no-relapse mortality after an allo-SCT [6]. aGvHD affects 40–72% of patients after allo-SCT. cGvHD is found in 30–70% of patients [7]. The clinical courses of both aGvHD and cGvHD are defined according to severity scores. Glucksberg et al. divide aGvHD into four grades, I-IV, of increasing clinical severity [8]. cGvHD is staged into 3 degrees of severity (mild, moderate, severe) as defined by the NIH Consensus Development Project, dated 2004 and 2014 [5,9], and multiple studies seem to prove the benefits of this score [10,11].

Clinical literature on GvHD predominantly focuses on prophylactic strategies, treatment modalities, or organ complications. Systematic analyses of the challenges, i.e., the physical and psychological symptoms patients are faced with, are scarce. To our knowledge, published data on aspects of supportive and palliative care, particularly in patients with severe aGvHD or cGvHD, have not been reviewed.

### 1.1. Severe Acute GvHD, as Defined by Grades III and IV

About 14% of patients that undergo allo-SCT suffer from aGvHD (grade III/IV), which we define as “severe aGvHD” for the scope of this review [12]. This definition includes all patients with severe symptoms affecting the skin, liver and (upper/lower) GI tract that correspond to a scoring system primarily used by Glucksberg et al. [8,13,14]. There are different risk factors for developing an aGvHD, with human leucocyte antigen disparity being implicated as the most important one [1,15,16]. Patients with more severe aGvHD often require higher doses of therapeutic immunosuppression [17]. With an increasing aGvHD grade, the response to immunosuppressive therapy tends to decrease, while organ involvement expands [15,18]. Furthermore, the predicted risk for and the development of aGvHD with the involved immunosuppressive prophylaxis and treatment significantly enhance the risk for fulminant and life-threatening viral and bacterial infections [19]. Severe aGvHD causes increased mortality [20], with a 30% probability of long-term survival for grade-III aGvHD and a long-term survival of less than 5% for grade-IV aGvHD [16].

### 1.2. Severe, Chronic GvHD

Following the 2014 NIH Consensus Criteria, severe cGvHD has been characterized by significant impairment of organ function. At least one of the relevant organs (skin, mouth, eyes, GI tract, liver, lungs, joints or fascia, or genital tract) is scored as severely affected (score of 3), or there is a lung score of 2 or 3. It can occur as a characteristic of cGvHD or as overlap syndrome of both aGvHD and cGvHD, with the latter being associated with a worse prognosis [5]. cGvHD, in its severe form, has a 2-year cumulative incidence that ranges from 8.3% to 27.6% [21]. It is a major cause of non-relapse mortality in patients surviving more than 2 years after allo-SCT. An increasing severity of cGvHD [6] and a progressive onset type of cGvHD [22] are associated with higher non-relapse mortality rates and lower overall survival as compared to milder forms of cGvHD [21]. Most cGvHD-related deaths are due to infection (60–85% of deaths in patients with cGvHD) or organ failures [6,23]. There are different risk factors for developing cGvHD, with prior aGvHD being the most important one [22,24]. The use of peripheral blood stem cells instead of bone marrow grafts is associated with a higher incidence of severe cGvHD [6,20]. The appearance of cGvHD might reduce the risk of relapse [22], but importantly, increased severity is not associated with a decreased relapse risk [6].

Patients who are usually allo-transplanted with a curative intent, but who are affected by severe GvHD, may suddenly find themselves in a life-threatening situation, potentially with a subsequent palliative trajectory [25]. This is a challenging situation for patients, their informal caregivers and health care professionals because all those involved in the allo-SCT procedure pursue a curative goal of care, with the expectation of long-term survival through this treatment modality [26]. The aim of this review is to summarize the current literature regarding the question whether patients with severe GvHD—as defined by aGvHD grade III/IV or severe cGvHD (according to NIH criteria)—might have special needs and burdens from the perspective of supportive and palliative care that require further investigation and treatment. To address these issues, outcomes like physical symptoms, quality of life (QoL) and psychological well-being are covered here. Since the integration of specialist palliative care in recipients of allo-SCT is currently under investigation and broadly supported [27], this review will additionally discuss the question if and at what point specialist palliative care should be included in standard-of-care procedures in patients with severe GvHD.

## 2. Materials and Methods

For this review, a focused, non-systematic literature search in the MEDLINE database via PubMed was performed in July 2020 with an update in October 2020. The following key words were searched for and combined with each other: gvhd, (severe) graft versus host disease, graft versus host reaction, palliative care, palliative care/medicine, palliative treatment, palliation, end of life care, hospice care, supportive care, advance care planning, psychooncology, psychological oncology, psychosocial oncology, long term survivors, quality of life (QoL), spiritual well-being, breathlessness, distress, pain. No restrictions on study design were applied and original articles as well as reviews were considered, based on their full text. Only publications in the English language were included. Further publications were identified by citation tracking, by means of the PubMed link ‘similar articles’ and cross-referencing from relevant publications.

For aGvHD, studies using the severity score of Glucksberg et al., or scoring systems based on it were included [8]. To distinguish between the different forms of severity of cGvHD, only studies that applied the 2004 and 2014 NIH Consensus Criteria were included [5,9]. Given the rarity of reports that specifically addressed supportive or palliative care needs in patients with GvHD, we also screened publications that more generally addressed aGvHD or cGvHD without specific distinction of severity grades. References to this kind of study are made clear in the subsequent text.

## 3. Results

We included 12 studies on acute or cGvHD that made a distinction between the different grades of severity of GvHD. Out of the 12 publications, only 1 focused on aGvHD. We did not identify any study that specifically examined patients with severe forms of GvHD.

### 3.1. Physical Symptoms and Functional Capacity

When considering symptoms in acute or cGvHD without differentiation of severity grade, it appeared that aGvHD was a risk factor for fatigue and sleep disturbance [28]. Active cGvHD seemed to be correlated with pain syndromes. Furthermore, cGvHD was a risk factor for fatigue and sexual dysfunction [29].

With regard to severe GvHD, the current literature does not provide sufficient information to describe possible unmet physical symptoms that are specifically related to a high severity of the disease. Only one cross-sectional study was identified, in which Im et al. examined the association between fatigue and severity of cGvHD in 263 patients (70.7% of study participants had severe GvHD) by use of the Lee Symptom Scale (LSS). This was a 30-item measure that was validated for evaluating adverse effects in cGvHD [30]. The authors did not find any negative association between the severity of GvHD and fatigue, although the symptom was prevalent in 84% of the included participants with cGvHD. Only a higher number of prior therapies for cGvHD showed a trend for an association with an increased incidence of fatigue [31].

With regard to physical function, a significant decrease of this parameter was shown in patients with aGvHD of all severity grades in a retrospective study by Hamada et al. However, compared to less severely affected patients, patients with severe aGvHD (15% of 76 study participants) had a longer time to recovery, which resulted in longer hospitalization. At discharge, physical functions in these patients were not fully restored, which was examined by measuring the knee extensor strength and performance of a 6-min walk test [32].

As for physical functions in cGvHD, Baird et al., in their cross-sectional natural history study, found worse outcomes in nearly all functional measures for patients with severe cGvHD (66% of 189 study participants) as compared to patients with milder forms of cGvHD [10]. To examine physical functioning, they used various patient-reported outcome measures (PROMs) such as LSS or the Short-Form-36 (SF-36), which is a 36-item survey with focus on mental and physical health and functioning. It includes a physical component summary measure (PCS) that combines the categories of physical functioning, role-physical and bodily pain [10,33]. PROMs allow to better measure the subjective outcomes of patients and to relate them to the NIH global score [34].

Their findings are in accordance with those by Pidala et al., who found that particularly the PCS was negatively affected in patients with severe cGvHD (39% of 567 study participants) [35]. In a multicenter, observational cohort study, they reported a significant association of an impaired 2-min walk test (2MWT) with higher symptom burden (measured by LSS), greater functional disability and more severe cGvHD (according to NIH global score) as well as more severe patient-reported cGvHD [35]. The 2MWT is an indicator for physiologic reserve and vulnerability, and its usefulness has been confirmed by Pavletic et al. in patients with cGvHD [36].

In addition, an association between a lower Karnofsky performance status (impaired functioning) of patients with cGvHD and higher cGvHD disease severity in several NIH sub-scores was shown. The Karnofsky performance status is a tool to measure disease burden and function [37].

In their systematic review with focus on QoL and functional capacity in patients with cGvHD, Agh et al. concluded that patients with more severe cGvHD had significantly lower functional capacities (measured by various PROMs) than patients with less severe cGvHD. In their opinion, GvHD symptoms could be seen as a predictor for functional capacity [38]. In contrast to most findings above, Mitchell et al. reported in a cross-sectional study with 100 study participants (50% with severe GvHD) that severity of cGvHD was not a predictor of functional performance [39].

An overview of the main results of our review on physical symptoms and physical functions in patients with severe GvHD is presented in Table 1.

### 3.2. Psychological and Spiritual Well-Being in Patients with Severe GvHD

#### 3.2.1. Psychological Well-Being

cGvHD, in general, is seen as a risk factor for persistent distress as well as for depression [29]. One-third of patients with cGvHD were reported to show significant depression or anxiety in a longitudinal observational study by Jacobs et al. In addition, the authors examined coping in patients with cGvHD, which was seen as an important factor for handling of stressful situations. They found task-oriented coping to be associated with fewer symptoms of depression and higher QoL over time in cGvHD. Avoidance in the form of social diversion also allowed better coping with cGvHD and showed better QoL as compared to other coping strategies. In this trial with 52 study participants, 71.2% had moderate, and 28.8% had severe cGvHD [40].

Little is known about the psychological well-being of patients with severe cGvHD. We identified only one secondary analysis of a prospective cohort that distinguished between the different grades of cGvHD, examining psychological distress by depression and anxiety. El-Jawahri et al. found that approximately 19.3% of 482 study participants with cGvHD suffered from depression, 22.8% had anxiety, and 14% showed symptoms of both, which was comparable with the findings of Jacobs et al. [40,41]. They could not find any association between the incidence of depression and the severity of cGvHD (40% had severe cGvHD), but patients with self-reported symptoms of depression showed a higher symptom burden of their cGvHD. Patients with self-reported anxiety had impairments in QoL and in physical functioning and also showed a higher GvHD symptom burden [41].

#### 3.2.2. Spiritual Well-Being

In a prospective longitudinal study that did not differentiate between cGvHD severity grades, Wong et al. named cGvHD to be the only important factor that significantly reduced spiritual well-being (Sp-WB) after allo-SCT in comparison to patients that did not suffer from cGvHD after allo-SCT [42].

Harris et al. could not find any effect of the severity of cGvHD on the level of spiritual well-being (Sp-WB) in a cross-sectional study. However, the longer patients suffered from cGvHD, the poorer Sp-WB was found to be. Sp-WB was assessed by the FACIT-Sp, which is a 12-item scale that measures meaning and purpose, harmony and peace, and closeness to God or to a higher power [43]. In this study, higher Sp-WB was positively associated with higher QoL. Out of the 51 participants included in this study, 4% had mild, 65% had moderate and 31% had severe cGvHD [43].

### 3.3. Quality of Life in Severe GvHD

In a multicenter, observational, prospective study, Pidala et al. were the first to evaluate whether severity of cGvHD, as defined by NIH criteria, was related to QoL as measured by the tools of SF-36 and the Functional Assessment of Cancer Therapy-Bone Marrow Transplant (FACT-BMT) [44]. They found disease severity to be the most significant determinant of impaired QoL independently of most other examined covariates such as other diseases or sociodemographic variables. In their study cohort of 298 participants, 10.4%, 58.7% and 30.9% had mild, moderate and severe cGvHD, respectively [44]. Baird et al. also found the NIH global severity score to significantly correlate with nearly all QoL PRO measures (including LSS, SF-36 PCS, FACT-BMT etc.). Furthermore, involvement of joints/fascia, sclerotic skin and lung disease showed the most significant impact on QoL [10]. Two additional cross-sectional studies examined the relation between QoL and NIH global severity and found similar results. In both, worse QoL was associated with worse NIH global severity of cGvHD. The first study had 1140 participants, with 9% suffering from severe cGvHD [45]. The second one included 264 participants, and 19.3% had severe cGvHD [46].

Pidala et al. further evaluated if changes in cGvHD severity by NIH global criteria were associated with changes in QoL, which could not be confirmed. Patient-reported changes in severity of cGvHD showed a much greater association with changes in all QoL measures as compared to clinician-reported changes and changes in GvHD severity as assessed by the NIH criteria. Apart from the global severity score, higher organ-specific severity grades also had a negative impact on QoL in patients with cGvHD [47]. According to Pidala et al., the organ-specific GI score and elevated bilirubin were also related to patient-reported QoL [48]. Agh et al. underlined higher severity of cGvHD to be a very important factor that negatively determined QoL in this patient group [38].

Hamilton et al. reported on the lower socioeconomic status of patients being associated with higher patient-reported severity of cGvHD and a reduced QoL, but they did not examine an association between severity of cGvHD, as objectified by the NIC global severity score, and socioeconomic status [49]. As for cGvHD without specification of severity, Jacobs et al. identified patients with cGvHD and lower perceived social support to experience poorer QoL [40]. An overview of the main results of our review on QoL in patients with severe GvHD is presented in Table 2.

Most of the retrieved data focuses on cGvHD, but Lee et al. found a measurable decline of QoL in patients with aGvHD in the first 6 months after allo-SCT, which improved within the first year post-transplantation, unless patients developed cGvHD [50].

## 4. Discussion

The appraisal of available literature for this review revealed a fundamental lack of large and representative studies describing supportive and palliative care needs of patients with severe GvHD. Publications that made clear assertions regarding aGvHD grade-III/IV or severe cGvHD were rare. However, we were able to extract some findings from general studies on GvHD, allowing us to draw first conclusions on palliative and supportive issues of patients suffering from the severe form of GvHD.

The literature findings suggest that patients with more severe GvHD suffer from worse physical functioning [10,32,35,38] and show a more impaired QoL as compared to those with less severe forms of GvHD [10,38,44,45,46]. With regard to physical symptoms, it was not possible to gather sufficient data to show if specific symptoms were especially burdensome in the severe forms of GvHD and if so, which ones those were. Nevertheless, the findings reported here indicated that special attention and treatment for those patients should be considered. Since psychological well-being can be impaired in patients with cGvHD [41], it is important to enhance research on the particular subset of patients suffering from severe GvHD following allo-SCT. The question should be addressed as to how these patients perceive their situation, what helps them to cope and which form of support should best be offered to them by whom (profession/discipline).

A main question is about the role that specialist palliative care (PC) can play for patients with severe aGvHD or cGvHD because severe GvHD may result in high symptom burden and a life-threatening situation [51]. In her review, Mitchell reports that severe GvHD could function as a longitudinal sentinel for the integration of specialist PC into standard care along the transplant trajectory [27]. In the large retrospective chart review, Han et al. found that the occurrence of GvHD after allo-SCT might be related to more frequent PC use [52]. El-Jawahri et al. surveyed transplant physicians about PC. Most of them named GvHD as a substantial unmet PC need after allo-SCT [53]. However, beyond these studies on PC after allo-SCT in general, we did not identify trials that specifically focused on the integration of specialist PC into the standard care of severe GvHD. This was quite surprising for us because patients who are treated in our medical center for severe forms of GvHD show a high level of burden and needs, requiring effective supportive and palliative care.

Due to our findings such as the high symptom burden, the loss of QoL and the highly impaired physical functions, as well as impaired psychological and spiritual well-being, we see the integration of PC into the care of patients with severe forms of GvHD as a main issue for clinical practice and for research [41]. Moreover, further important topics of PC such as communication about prognosis and end-of-life matters, prognosis perception, quality of end-of-life care and advance care planning also need to be considered. Research in this field is highly warranted.

We can only hypothesize about the reasons for the lack of such data on severe GvHD because at least in our medical center we perceive this patient group to have a high level of suffering and very special needs, which warrants intensified research. The burden of disease could, in fact, be a great impediment that might cause this shortage of systematic data because it might be difficult to include patients with severe GvHD in studies due to their instable medical condition and reduced physical and cognitive function [35]. Since severe GvHD causes high morbidity, patients might die prior to completion of a study [54] or before a study even takes place. Those circumstances require special study designs in this area, and to examine this patient group, studies should focus on severe GvHD patients directly.

Furthermore, rating the severity of GvHD only by taking into consideration the NIH global severity score might be insufficient. Although some trials show a good correlation of the scores and outcomes such as QoL, patients with GvHD form a very heterogenous population and might undergo rapid changes in morbidity. For this reason, integration of PROMs as an integral endpoint into research and treatment of GvHD is of high interest. Such PROMs allow the evaluation of issues that go beyond the clinician’s perspective of severity and should also be considered for our patient group with severe GvHD [55,56].

## 5. Conclusions

This review shows a fundamental lack of representative studies to describe the burden and needs of patients with severe GvHD after allo-SCT and the role of supportive and palliative care for this patient group. Findings in patients with severe GvHD, such as a more impaired QoL and reduced physical functioning as compared to patients with less extensive and less severe GvHD, indicate that research in this field is highly warranted. Aiming to integrate specialist PC early, it is important to examine at what point specialist PC should be integrated into the allo-SCT trajectory, particularly from the patient’s point of view. We consider this review to promote awareness and act as a primer for such initiatives. Further research is needed to be able to draw a comprehensive and differentiated picture of the burden, needs and resources of affected patients and to optimize their treatment with a multidisciplinary approach.

## Figures and Tables

**Table 1:** Publications identified by the PubMed search on physical symptoms and functions in patients with GvHD, with specific results on severe GvHD.

First Author, Year, Country	Study Design	Form of GvHD	Patient Population	Main Outcomes	Main Results on Severe GvHD
Im, 2016, USA [31]	Cross-sectional	cGvHD	263 study participants; 70.7% had severe cGvHD	Fatigue in patients with moderate to severe cGvHD	84% of participants endorsed fatigue in different forms, but no correlation was found between the prevalence of fatigue and NIH cGvHD severity.
Hamada, 2019, Japan [32]	Retrospective chart review	aGvHD	76 study participants; 15% had aGvHD grades III and IV	Effect of the severity of aGvHD on physical function	A decline in physical functions was shown for all participants who developed aGvHD, but participants with aGvHD grades III and IV showed worse recovery of physical functions compared to participants with milder forms.
Baird, 2013, USA [10]	Cross-sectional	cGvHD	189 study participants; 66% had severe cGvHD	Assessment of the validity of the NIH criteria as determinants in severely affected patients	Participants with more severe cGvHD showed worse outcomes in nearly all functional measures compared to milder forms of cGvHD.
Pidala, 2013, USA [35]	Prospective cohort	cGvHD	584 study participants; 39% had severe cGvHD	Assessment of physical functions in patients with cGvHD by HGS and 2MWT and its relation to PROs	A significant association of an impaired 2MWT with higher symptom burden (measured by LSS), greater functional disability and more severe cGvHD was shown.
Agh, 2019, Hungary [38]	Systematic Review	cGvHD	n.a.	Systematic overview of HRQoL and functional capacity of patients with cGvHD	More severe cGvHD was correlated with worse physical functions.
Mitchell, 2009, USA [39]	Cross-sectional	cGvHD	100 study participants; 50% had severe cGvHD	Assessment of determinants of functional performance in patients with cGvHD	cGvHD severity was no significant predictor of functional performance.

aGvHD: acute graft-versus-host disease; cGvHD: chronic graft-versus-host disease; HGS: hand grip strength; HRQoL: health-related quality of life; LSS: Lee symptom scale; 2MWT: 2-min walk test; n.a.: not applicable; NIH: National Institutes of Health; PROs: patient-reported outcomes.

**Table 2:** Publications identified by the PubMed search on quality of life in patients with GvHD with specific results on severe cGvHD.

First Author, Year, Country	Study Design	Form of GvHD	Patient Population	Main Outcomes	Main Results on Severe GvHD
Pidala, 2011, USA [44]	Cross-sectional	cGvHD	298 study participants; 30.9% had severe cGvHD	Association of patient-reported QoL and the severity of cGvHD	Severe cGvHD was associated with worse patient-reported QoL compared to milder forms of cGvHD.
Baird, 2013, USA [10]	Cross-sectional	cGvHD	189 study participants; 66% had severe cGvHD	Assessment of the validity of the NIH criteria as determinants in severely affected patients	Participants with more severe cGvHD showed worse outcomes in nearly all QoL measures compared to milder forms of cGvHD.
Kurosawa, 2017, Japan [45]	Cross-sectional	cGvHD	1140 study participants; 9% of patients had severe cGvHD	Association of patient-reported QoL and the severity of cGvHD	Severe cGvHD was associated with worse patient-reported QoL compared to milder forms of cGvHD.
Mo, 2013, China [46]	Cross-sectional	cGvHD	264 study participants; 19.3% had severe cGvHD	Association of patient-reported QoL and the severity of cGvHD	Severe cGvHD was associated with worse patient-reported QoL compared to milder forms of cGvHD.
Pidala, 2011, USA [47]	Prospective cohort	cGvHD	336 study participants; 27% had severe cGvHD at the beginning of the study	Association of changes in cGvHD severity (measured by NIH criteria by the clinician as well as patient-reported)	Changes in NIH-assessed cGvHD severity did not have an impact on changes in QoL, but changes in patient-reported cGvHD severity were associated with changes in QoL measures.
Agh, 2019, Hungary [38]	Systematic review	cGvHD	n.a.	Systematic overview of HRQoL and functional capacity of patients with cGvHD	Four of five included studies that assessed the impact of cGvHD severity on HRQoL found significantly worse HRQoL in patients with more severe cGvHD.

cGvHD: chronic graft-versus-host disease; HRQoL: health-related quality of live; n.a.: not applicable; NIH: National Institutes of Health; QoL: quality of life.
